# Social Media Recruitment in Indigenous and Native American Populations: Challenges in the AI Age

**DOI:** 10.2196/76677

**Published:** 2025-09-17

**Authors:** Nadia Diamond-Smith, Alison Comfort, Anna Epperson, Alicia R. Riley, Natalie Beylin, Mary Garcia, Sarah Francis, Lucía Abascal Miguel

**Affiliations:** 1 Institute for Global Health Sciences, University of California, San Francisco, 550 16th St, San Francisco, CA, 94117, United States; 2 Department of Epidemiology and Biostatistics, University of California, San Francisco, San Francisco, United States; 3 Department of Obstetrics, Gynecology and Reproductive Sciences, University of California, San Francisco, San Francisco, United States; 4 Health Sciences Research Institute, University of California Merced, , Merced, CA, United States; 5 Department of Psychological Sciences, University of California Merced, Merced, CA, United States; 6 Sociology Department, University of California, Santa Cruz, Santa Cruz, United States; 7 UpSwell, Seattle, WA, United States

**Keywords:** social media recruitment, indigenous populations, Native American populations, public health research, survey design, data quality

## Abstract

Using social media recruitment for public health research presents both opportunities and challenges. Despite its increased use, few studies have detailed the practical issues, challenges encountered, and alternative strategies available for social media recruitment. This paper explores strategies for recruiting Indigenous and Native American populations in California for a study on COVID-19 vaccination and social networks. We describe different recruitment approaches, challenges faced, and pros and cons of strategies used to enhance data quality and efficiency, including survey design considerations, Facebook targeting versus use of research panels, quality assurance checks, and decisions around participant incentives. Our local setting involved recruiting Native American and Mesoamerican Indigenous individuals living in California through social media platforms. We highlight key adaptations to survey design, recruitment strategies, and data cleaning processes, noting what approaches that were effective and those that were not. Despite targeted efforts and community collaboration, recruitment was limited, and fraudulent data from bots significantly compromised data quality. Standard Facebook targeting approaches were largely unsuccessful. Our findings suggest that the increasing sophistication of artificial intelligence is becoming a substantial obstacle to authentic participant recruitment through social media. We offer recommendations to improve recruitment of hard-to-reach populations and mitigate AI-related fraud risks in future research.

## Introduction

Native American and Indigenous populations have historically been underrepresented in health research due to longstanding structural, geographic, and cultural barriers, including limited trust in research institutions, language barriers, digital access challenges, and lack of culturally tailored outreach [1,2]. The rise of social media has sparked interest in its use for recruiting participants in both observational studies and online intervention evaluations. While most research focuses on social media to recruit for interventions, fewer have used it for observational research [3,4]. Social media has been promoted as an effective method for recruiting “hard-to-reach” populations in high-income countries, particularly sexual and gender minorities, with some studies examining effectiveness, cost-efficiency, and representativeness, and some providing guidance on specific approaches or challenges [5-9].

Despite the expansion of digital tools during the COVID-19 pandemic, few studies have described the use of online methods to engage Native American or Mesoamerican Indigenous communities. Some efforts have explored web-based health promotion or survey research with tribal communities, often emphasizing the importance of tribal sovereignty, trust-building, and culturally grounded methods [10,11]. However, few papers have systematically reported the methodological challenges, outcomes and lessons learned of social media recruitment in these population. Moreover, the rise of advanced artificial intelligence (AI) technology has exacerbated these challenges due to the proliferation of bots and fraudulent entries. Thus, there is a gap in practical guidance for researchers aiming to use social media as a recruitment tool, particularly for hard-to-reach populations in the age of AI.

In this “Notes from the Field,” we report our experience recruiting Native American and Mesoamerican Indigenous populations through social media in California for a study examining COVID-19 vaccination and social networks. The parent study aimed to recruit 800 Native American and Mesoamerican Indigenous individuals living in California, as well as 800 participants who were racially and ethnically representative of California’s general population. Between January 2024 and February 2025, we recruited a total of 1173 participants, including 209 Native American and Mesoamerican Indigenous individuals. Recruitment approaches included Facebook and Instagram advertisements, outreach through community partner (CBOs), and use of a preformed research panel from Qualtrics (Table 1). Our survey included 50 questions, took approximately 30 minutes to complete, and used validated measures assessing social networks, vaccine attitudes, social media use, and sociodemographic variables. We pilot-tested the tool with students and members of our Indigenous Community Advisory Board to ensure cultural relevance and clarity, and was subsequently revised based on the feedback. In total, we received 5099 survey responses from the general population, of which 3926 were excluded due to suspected poor quality or fraudulent data. Our final sample included 137 (12%) Native American and 72 (6%) Mesoamerican Indigenous participants, far from our initial goal.

**Table 1.** Recruitment strategy and output.

Recruitment strategy	Incentive	Responses collected (N=3382)	Responses kept (n=1173)	NA/AI^a^ (n=137)	Indigenous (n=72)
Meta ads - general incentive	$30	1212	334 (27%)	39	3
Meta ads - Raffle	Raffle	246	35 (14%)	2	1
Meta ads with Facebook Messenger screener	Raffle	315	95 (30%)	5	5
Qualtrics preformed panel	Set by them	1518	662 (44%)	76	49
CBO^b^ distribution	$30	91	47 (52%)	15	14

a NA/AI: Native American/ American Indian.

b CBO: community-based organization.

Study results were used to examine how social networks influence vaccine decision-making and to inform the design of an intervention to address vaccine hesitancy among these subgroups. This study received institutional review board approval from participating universities and the California Rural Indian Health Board (UCSF IRB #23-38709, CRIHB IRB #2024-003) . It was also guided by a Community Advisory Board composed of Native American and Mesoamerican community members. The lead research team includes individuals from these communities.

This “Notes from the Field” highlights what was effective and what fell short in our methods and approach, and offers recommendations on recruitment strategies, survey design, and post-data collection quality assurance for researchers interested in utilizing social media for recruiting racial and ethnic minority groups in the United States.

## Lessons Learned

### Survey Design

We designed a survey suitable for a small screen, recognizing that some participants would complete it on mobile devices with limited time [12]. Pilot testing indicated that longer surveys increased dropout rates, prompting us to limit survey completion time to under 20 minutes. Additionally, answer options requiring extensive scrolling caused participant disengagement. To improve retention, we adapted the survey by using concise questions that fit entirely on a single screen.

Based on prior experience, we anticipated the risk of bots submitting fraudulent data to claim incentives and took proactive measures to mitigate this issue. We implemented several “bot-catching” quality checks, which also helped identify disingenuous participants rushing through the survey. Many online survey platforms offer built-in tools to verify human participants, such as CAPTCHAs, a screening mechanism where participants complete easy tasks but are challenging to bots and “invisible” reCAPTCHA’s, which humans bypass unnoticed but are designed to trap bots. Recent studies have shown that they are increasingly vulnerable to sophisticated bots and human-assisted workarounds, making them a necessary but insufficient defense when used alone [13]. In our survey, many responses were dropped due to failed CAPTCHA verification; however, approximately 70% (n= 2367/3382) of the responses ultimately deemed ineligible had successfully passed the CAPTCHA screen.

We also experimented with “Honeypot” questions, which are questions that try to trick chatbots or insincere participants rushing who may not be reading the question but simply providing responses. These questions prompted respondents to select a specific answer (eg, the third option). However, we found that bots were able to bypass these as well. Ultimately, open-response fields, such as “other” with a fill-in-the-blank option or short-answer questions, proved more effective. These fields allowed us to assess whether responses were rational and relevant, to help confirm participant authenticity.

After designing the survey, the next step was programming it using Qualtrics, an electronic survey platform, to integrate screeners, consent forms, and the survey itself. These platforms generate sharable links, making it easy to distribute surveys via social media. However, one drawback is that participants are redirected to an external webpage, often causing drop-offs. A potential workaround is embedding the survey directly within platforms like Facebook Messenger. However, this imposes limitations on the types of questions and branching logic that can be used, requiring simpler formats as these platforms are not specifically designed for complex surveys.

The screening process varied across recruitment strategies. When participants were recruited through Facebook and Instagram and directed to Qualtrics, the screener appeared as the first set of survey questions and included items on residence, age, and race and ethnicity (the race/ethnicity item was omitted when recruitment was open to the general population). Using this approach, only 33 of the 1,212 responses received were deemed ineligible based on the screener. In the Messenger-based approach, participants first completed a brief screener embedded within Facebook Messenger. Those who met eligibility criteria were then provided a link to the Qualtrics survey, which began with the consent form. Approximately 100 individuals were screened using this method, with half (n=50) determined ineligible. For the panel recruitment, we provided eligibility criteria to the panel provider (Qualtrics), who prescreened participants and invited only eligible individuals to participate. These participants were directed straight to the survey’s consent form. Across all recruitment strategies, approximately 18 individuals did not consent to participate after reaching the survey.

### Recruiting

Social media platforms offer advanced advertising tools that have transformed recruitment strategies. For instance, Meta’s Ads Manager allows for efficient creation, monitoring, and optimization of campaigns. One major benefit of using Meta ads is the ability to implement highly targeted recruitment. Campaigns can focus on broad geographic regions or pinpoint specific areas, such as a single zip code. Additionally, targeting criteria can be refined based on factors like language, age, gender, and specific interests, allowing researchers to precisely reach their intended audience.

Using Meta’s advertising tools, we targeted recruitment to zip codes with high concentrations of our target population. In collaboration with our community advisory board (CAB), we refined targeting criteria to include interests we hypothesized would resonate with the population, such as the Mexican national soccer team and Ranchera music for individuals with Mexican heritage. Images for ads were selected in partnership with the CAB and with insights from Upswell, they varied depending on the target audience. Campaigns were divided by demographics so we could turn off when we had reached the recruitment target. The objective of the campaign was survey clicks and the ads were active for two-week periods over four months. However, this approach yielded low recruitment rates. A more strategic selection of target interests, informed by deeper community insights or additional testing, could have improved this approach.

To enhance recruitment, we next employed a preformed panel—a curated group of individuals who had previously agreed to participate in research studies. We chose Qualtrics for this purpose, as our survey was already designed within their platform, and our university maintained an existing partnership. While this approach required significant upfront costs (and initial payment to Qualtrics), it proved more cost-effective compared to social media in the end in terms of cost per participant recruited. After filtering out suspected fraudulent responses, we found that recruitment through Meta ads cost approximately 10 times more than using the Qualtrics panel per high quality (real) participant recruited. In addition to cost benefits, the panel led to much faster recruitment compared to social media. However, given that our focal population included hard-to-reach groups (especially the Mesoamerican Indigenous population living in California), the pool of eligible individuals within the panel was quickly exhausted.

### Incentives and Suspected Fraudulent Data

Participant compensation was a critical aspect of our recruitment strategy. Initially, based on community partner recommendations, we offered a $30 USD gift card to all participants. However, this led to a high volume of suspected fraudulent data, with 73% (n=884) of responses discarded during the initial data collection phase due to bots and fake submissions. To address this issue, we transitioned to a raffle system, offering a US $100 prize to 1 in 50 respondents. However, recruitment remained limited and fraudulent entries persisted—even after further improving the raffle odds to 1 in 10, recruitment levels did not increase and we continued to encounter suspected fraudulent data.

To balance recruitment needs with data quality, we implemented a system of individual, one-time survey links for each participant. These links could only be used once by one person, and thus, could not be shared or used multiple times over by a chatbot. Participants still received the link through a social media ad, but it could not be circulated further. This allowed us to offer a higher incentive while controlling access to prevent bots and fake entries. However, this method was labor-intensive for both our team and the participants. Interested individuals were required to email or text us to request access, after which we manually provided them with a survey link. While effective in improving data authenticity, this process limited our potential reach. We focused recruitment efforts on controlled channels, including Facebook and WhatsApp groups managed by our community partners, as well as direct contacts. While these methods helped ensure the authenticity of participants, they presented several challenges. Many individuals in our target population did not use email, and the delay between participants expressing interest and receiving their unique survey link further reduced follow-through.

During data collection, we encountered a high volume of suspected fraudulent entries, identified through re-CAPTCHA scores and other quality checks. Many fraudulent entries bypassed these measures in place by Qualtrics, requiring us to conduct extensive manual data review and cleaning to ensure sample quality. To address these challenges, we reprogrammed the survey screener to run through Facebook Messenger. When users clicked on the ad, it opened an automated chat that included the survey screener, and eligible participants were then provided with the survey link. While this approach reduced bot activity, it came with trade-offs: overall participation decreased, the process was less cost-effective, and the platform’s interface presented additional challenges.

Following data collection, we began a systematic data-cleaning process to ensure sample quality. We excluded all responses with a bot likelihood score below 0.5 provided by Qualtrics. Furthermore, we examined the geolocation of respondents’ IP addresses to ensure they met our eligibility criteria, discarding those outside our target area. We also removed duplicate IP addresses; however, this process also carried the possibility of excluding legitimate responses from multiple participants within the same household.

To further improve data quality, we applied time filters to discard responses completed too quickly (ie, beyond two standard deviations from the mean). Additionally, collecting email addresses to distribute gift cards provided another opportunity for verification. We manually reviewed these emails for authenticity, flagging suspicious entries characterized by random strings of numbers or letters (eg, la675829@gmail.com).

## Conclusions

Our experience with recruitment through social media revealed two key lessons. First, while social media has been effective for recruiting general populations and some specific subgroups, such as sexual and gender minorities, this approach was challenging in engaging our study’s hard-to-reach populations—Mesoamerican Indigenous and Native American individuals in California. This was true despite the study team’s prior experience working with these populations, expertise in social media recruitment, and input from a community advisory board on survey design and recruitment strategies. Language barriers, as our survey was only available in Spanish and English, may have contributed to these difficulties, highlighting the need for more culturally and linguistically tailored approaches to recruitment.

Second, the prevalence of suspected fraudulent data was more difficult to address than our team’s prior experiences collecting data via social media in both the United States and globally. Sophisticated bots are capable of bypassing traditional quality checks, highlighting a need for strategies to differentiate authentic participants from automated responses. Identifying and filtering out fraudulent data required significant time and resources, increasing costs. Thus, it is possible that the age of easy, fast, inexpensive research through social media may be waning with increasingly sophisticated AI developments. To sustain the social media as a beneficial research tool, novel approaches to detect and prevent fraudulent entries are critically needed.

## Abbreviations

AI: artificial intelligence
CAB: community advisory board
CBO: community-based organization
